# Integrated Membrane Process in Organic Media: Combining Organic Solvent Ultrafiltration, Nanofiltration, and Reverse Osmosis to Purify and Concentrate the Phenolic Compounds from Wet Olive Pomace

**DOI:** 10.3390/ijms25105233

**Published:** 2024-05-11

**Authors:** Carmen M. Sánchez-Arévalo, Fausto Aldegheri, M. Cinta Vincent-Vela, Silvia Álvarez-Blanco

**Affiliations:** 1 Research Institute for Industrial, Radiophysical and Environmental Safety (ISIRYM), Universitat Politècnica de València, Camino de Vera s/n, 46022 Valencia, Spain; carsana5@upv.es (C.M.S.-A.); fausto.alde@gmail.com (F.A.); mavinve@iqn.upv.es (M.C.V.-V.); 2 Department of Chemical and Nuclear Engineering, Universitat Politècnica de València, Camino de Vera s/n, 46022 Valencia, Spain

**Keywords:** phenolic compounds, organic solvent ultrafiltration, organic solvent nanofiltration, reverse osmosis, integrated process, ethanol

## Abstract

Phenolic compounds from a hydroalcoholic extract of wet olive pomace were purified and concentrated by an integrated membrane process in organic media. First, UF010104 (Solsep BV) and UP005 (Microdyn Nadir) membranes were tested to be implemented in the ultrafiltration stage, with the aim of purifying the extract and obtaining a permeate enriched in phenolic compounds. Despite the high flux observed with the UF010104 membrane (20.4 ± 0.7 L·h^−1^·m^−2^, at 2 bar), the UP005 membrane was selected because of a more suitable selectivity. Even though some secoiridoids were rejected, the permeate stream obtained with this membrane contained high concentrations of valuable simple phenols and phenolic acids, whereas sugars and macromolecules were retained. Then, the ultrafiltration permeate was subjected to a nanofiltration step employing an NF270 membrane (DuPont) for a further purification and fractionation of the phenolic compounds. The permeate flux was 50.2 ± 0.2 L·h^−1^·m^−2^, working at 15 bar. Hydroxytyrosol and some phenolic acids (such as vanillic acid, caffeic acid, and ferulic acid) were recovered in the permeate, which was later concentrated by reverse osmosis employing an NF90 membrane. The permeate flux obtained with this membrane was 15.3 ± 0.3 L·h^−1^·m^−2^. The concentrated phenolic mixture that was obtained may have important applications as a powerful antioxidant and for the prevention of diabetes and neurodegenerative diseases.

## 1. Introduction

Phenolic compounds attract enormous attention nowadays because of their numerous applications in the pharmacological industry, the food industry, and even in the beauty market [1,2,3]. One of the main attributes of these compounds is their strong antioxidant capacity, which is linked with numerous health benefits. For some time now, the interest in obtaining these health benefits from natural sources has grown [4]. This trend is based on the attempt to pursue holistic wellness and preventive healthcare.

As previously demonstrated, the phenolic compounds that are naturally existing in some vegetal products have been related to powerful antioxidant, antimicrobial, and anti-inflammatory capacities [5,6,7,8]. As consumer awareness continues to expand and scientific evidence supporting the benefits derived from phenolic compounds accumulates, the trend towards embracing these natural compounds is likely to persist in the years to come. Therefore, it is essential to find adequate sources for these valuable biomolecules.

They can be found in natural sources such as fruits and vegetables [9]. Interestingly, these valuable compounds are also present in many byproducts generated by the agri-food sector, such as industries of olive oil, wine, orange, artichoke, avocado or tea [10,11,12,13,14]. Employing these by-products as a source of high added-value compounds can contribute to the circular economy of many industries, increasing their sustainability. Furthermore, it may transform a residue into a resource, capable of providing a profit for industrialists. With this practice, the incorrect disposal of these residues and any subsequent environmental impact could eventually be avoided. At the same time, a plethora of antioxidant compounds with valuable health properties can be obtained.

In the context of the olive oil industry, which is one of the most important food industries in the Mediterranean area [15], wet olive pomace is an excellent candidate to be used as a natural and cheap source of phenolic compounds. This by-product is abundantly generated during olive oil production when the so-called two-phase methodology is applied [16]. According to the two-phase methodology, olive oil is separated from the vegetation water and the solid fraction from the olive fruit by means of centrifugation. No water is added during this stage (in contrast with the alternative three-phase methodology). As a result, in processing 1000 kg of olives, virgin olive oil is obtained (200 kg), and, at the same time, 800 kg of wet olive pomace is produced, approximately.

Wet olive pomace contains the remnants of the olive fruit after extracting the oil; therefore, it consists of the olive skin, pulp, stones, and seeds, as well as the vegetation water. Consequently, a considerable proportion of the olive minor fraction remains in wet olive pomace [17]. This fraction entails a group of compounds that are not abundant in the olive fruit, but they are very significant due to their bioactivities. Among these compounds, triterpenes, lignans, phytosterols, tocopherols, free fatty acids, and the valuable phenolic compounds can be found [18].

The recovery of the phenolic compounds from wet olive pomace can be accomplished using solid–liquid extraction to extract the compounds from the solid matrix, followed by the application of membrane technology in order to purify and, when necessary, concentrate the molecules. In fact, the advantages of membrane technology to treat and valorize agri-food by-products have been previously described [19,20]. However, the vast majority of the contributions in this regards deal with aqueous streams [21,22].

When a solid residue, such as wet olive pomace, is employed, the efficiency of the extraction highly influences the overall recovery of the process, as it defines the initial concentration. During the subsequent membrane processes, a proportion of the target compounds will inevitably be lost. Therefore, maximizing the initial concentration of the compounds in the extract to be treated is of relevance. When the extraction process is performed with a mixture of ethanol/water 50:50 (*v*/*v*), a higher concentration of biophenols is obtained, in comparison with a water-based extraction. This mixture ensures an adequate polarity for the medium to withdraw the phenolic compounds from the vegetal matrix [23]. However, the membrane processes downstream the hydroalcoholic extraction are normally much more challenging because the presence of an organic solvent in the feed solution introduces an additional feature to be considered. The permeate flux might be reduced, the rejection values might be unexpected, and even the membrane integrity can be compromised [24,25]. For these reasons, despite the benefits in terms of concentration, when valuable compounds are recovered from agri-food residues by membrane technology, the previous extraction of such compounds is typically water-based. This was the case for Nunes et al. [26], who applied nanofiltration and reverse osmosis to an aqueous extract of wet olive pomace, and Tapia-Quirós et al. [27], who studied several membrane processes, including microfiltration, ultrafiltration, nanofiltration and reverse osmosis, to recover polyphenols from olive pomace after an extraction with water. Sygouni et al. [28] concluded that solvent-extraction, employing ethanol at 50% (*v*/*v*), of the biophenols from wet olive pomace was the most efficient; however, they proceeded to the membrane process with the aqueous extract.

Only a few contributions dealing with membrane filtration of a hydroethanolic extract of wet olive pomace have been published to date. In a previous study by the authors, the potential of ultrafiltration to reduce the organic load of the hydroalcoholic wet olive pomace extract was demonstrated, while the phenolic compounds were purified in the permeate [29]. Later, the authors also studied the performance of several nanofiltration membranes to treat a hydroalcoholic model solution whose composition was based on the extract of wet olive pomace obtained with ethanol/water 50:50 (*v*/*v*) [30]. However, the development of a solvent-mediated, integrated process, covering all the steps from the extraction of the phenolic compounds to their purification by membrane technology, and their final concentration, is missing in the literature.

Therefore, in this contribution, a combination of ultrasound-assisted solid–liquid extraction (with the solvent ethanol/water 50:50 (*v*/*v*)) with ultrafiltration, nanofiltration, and reverse osmosis has been proposed for the first time, aiming to obtain a final concentrate of purified phenolic compounds from wet olive pomace.

## 2. Results

### 2.1. Characterization of the Hydroalcoholic Extract of Wet Olive Pomace

The extract of wet olive pomace was obtained with ethanol/water 50:50 (*v*/*v*), according to a previously investigated process [23]. The volume of extract obtained by this methodology was 8 L. It was characterized prior to its utilization in the ultrafiltration plant. The characteristics of this extract can be found in Table 1.

### 2.2. Organic Solvent Ultrafiltration

#### 2.2.1. Productivity of the Process

**Figure 1:**
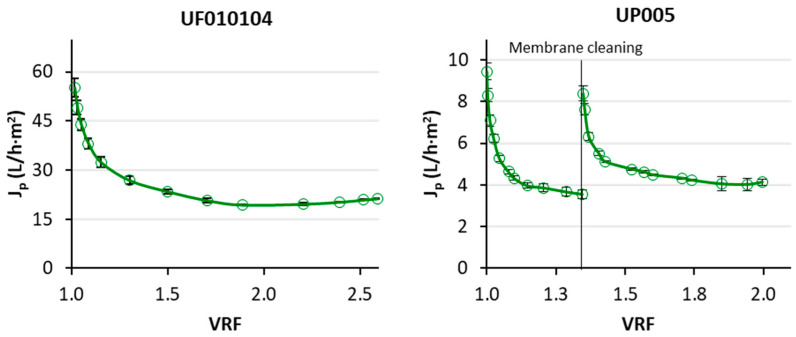
Permeate flux values obtained when a hydroalcoholic extract of wet olive pomace was treated with the UF010104 and UP005 membranes at 1.8 m/s and 2 bar and 1.8 m/s and 2.5 bar, respectively.

To assess the productivity of the ultrafiltration step, the permeate flux was measured. The UF010104 membrane displayed a solvent permeability of 72.2 L·h^−1^·m^−2^·bar^−1^, whereas the solvent permeability of the UP005 membrane was 8.0 L·h^−1^·m^−2^·bar^−1^. This difference was expected, based on the distant molecular weight cutoff (MWCO) of both membranes. The values of permeate flux obtained when the ethanolic extract of wet olive pomace was ultrafiltered are reflected in Figure 1.

#### 2.2.2. Rejection Values Obtained in the Ultrafiltration Stage

##### Rejection of Undesired Organic Matter

**Figure 2:**
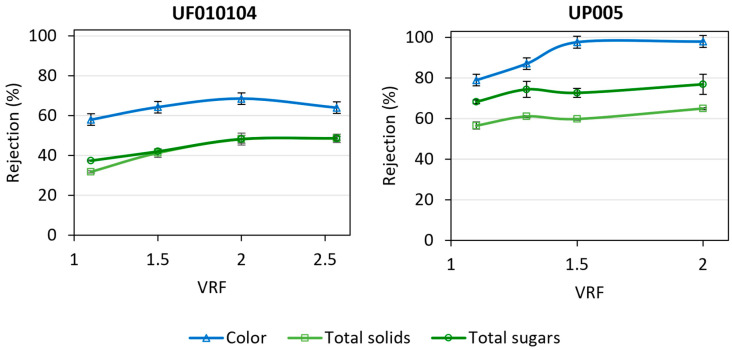
Evolution of the rejection of color, total solids, and total sugars with volume reduction factor (VRF) during the ultrafiltration of a hydroalcoholic extract of wet olive pomace. On the left side: results for the UH010104 membrane at 1.8 m/s and 2 bar. Right side: results for the UP005 membrane at 1.8 m/s and 2.5 bar.

In order to purify the extracted phenolic compounds, a high rejection of the concomitant organic matter was intended. Figure 2 contains the rejection of color, total solids, and sugars obtained with the tested ultrafiltration membranes.

##### Rejection of Phenolic Compounds

**Figure 3:**
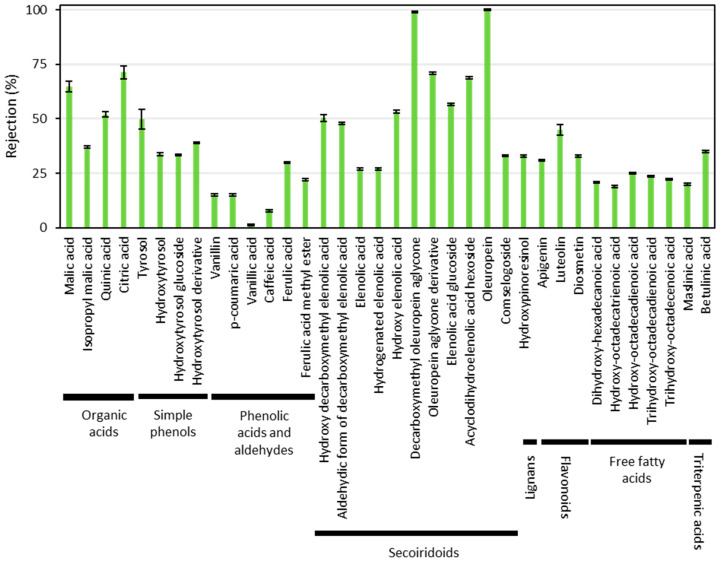
Individual rejection of each metabolite detected by LC-MS after the ultrafiltration of the hydroalcoholic extract of wet olive pomace with the UP005 membrane at a volume reduction factor of 2. The operating conditions were 1.8 m/s and 2.5 bar.

According to our preliminary studies [23], a significant proportion of the phenolic content of the extract of wet olive pomace is recovered in the permeate of the UP005 membrane. To achieve a thoughtful knowledge into the rejection of each individual compound, the samples were characterized by liquid chromatography coupled to quadrupole-time-of-flight mass spectrometry, using an electrospray interphase (LC-ESI-QToF-MS). This characterization allowed us to determine 36 compounds belonging to the olive minor fraction. Among them, eight chemical families were identified. Based on their elution order from the chromatographic column, these families were organic acids, simple phenols, phenolic acids and aldehydes, secoiridoids, lignans, flavonoids, triterpenic acids, and free fatty acids. The organic acids included quinic acid, citric acid, malic acid, and isopropyl malic acid. These are compounds usually found in vegetal extracts. In this case, they were non-desired, as they contribute to reduce the purity of phenolic compounds. This was also applicable to free fatty acids, which resulted from the degradation of the olive triacylglycerides. They included trihydroxy-octadecadienoic acid, trihydroxy-octadecenoic acid, dihydroxy-hexadecanoic acid, hydroxy-octadecatrienoic acid, and hydroxy-octadecadienoic acid. The chemical class of simple phenols contained tyrosol, hydroxytyrosol, and hydroxytyrosol derivatives (glucosides, mainly). Among the phenolic acids and aldehydes, vanillic acid, caffeic acid, *p*-coumaric acid, ferulic acid, ferulic acid methyl ester, and vanillin were found. The secoiridoid acids entailed compounds such as acyclodihydroelenolic acid hexoside, hydroxy decarboxymethyl elenolic acid, hydroxy elenolic acid, elenolic acid glucoside, oleuropein aglycone derivative, decarboxymethyl elenolic acid and its aldehydic form, hydrogenated elenolic acid, comselogoside, elenolic acid, hydroxypinoresinol, oleuropein, and decarboxymethyl oleuropein aglycone. Only one lignan, hydroxypinoresinol, was found. Among the flavonoids, luteolin, apigenin, and diosmetin were present in the samples. Finally, the chemical family of triterpenic acids included maslinic acid and betulinic acid. The individual rejection of all these compounds is presented in Figure 3.

### 2.3. Organic Solvent Nanofiltration

The permeate stream obtained during the ultrafiltration step was submitted to a nanofiltration process, employing the NF270 membrane, whose solvent permeability was 5.08 L·h^−1^·m^−2^·bar^−1^. In a previous study from our research group, a screening of numerous commercial membranes to assess the organic solvent nanofiltration of a synthetic extract of wet olive pomace was performed [30]. In that study, the NF270 membrane stood out because of a high permeate flux and an efficient fractionation of phenolic compounds, as well as their separation from sugars. According to the good results obtained during the treatment of the simulated solution, the NF270 membrane was selected in this work to treat a real hydroalcoholic extract of wet olive pomace after its ultrafiltration.

**Figure 4:**
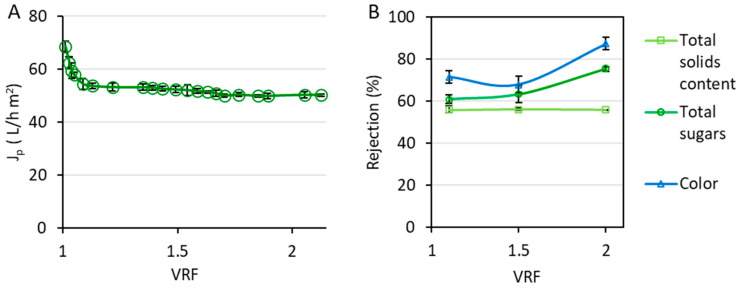
Permeate flux (**A**) and rejection of total sugar content, total solids, and color (**B**) obtained during the nanofiltration stage with the NF270 membrane at 1 m/s and 15 bar.

The permeate flux obtained during the nanofiltration of the hydroalcoholic permeate obtained during the ultrafiltration process is reflected in Figure 4A. Figure 4B shows the rejection values, regarding the unwanted organic matter, achieved with the NF270 membrane. Furthermore, the total rejection coefficient for the integrated process until this point (after the ultrafiltration and nanofiltration stages) was 94 ± 9% and 82.73 ± 0.05% for total sugars and total solids, respectively.

**Figure 5:**
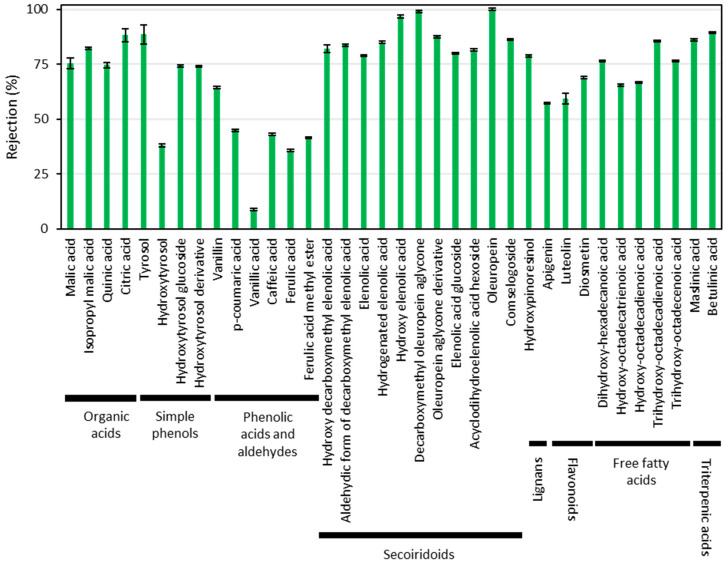
Individual rejection of each metabolite detected by LC-MS after the nanofiltration of the hydroalcoholic ultrafiltration permeate with the NF270 membrane at a volume reduction factor of 2. The operating conditions were 1 m/s and 15 bar.

To address the rejection of phenolic compounds by the NF270 membrane, Figure 5 is provided.

### 2.4. Reverse Osmosis

**Figure 6:**
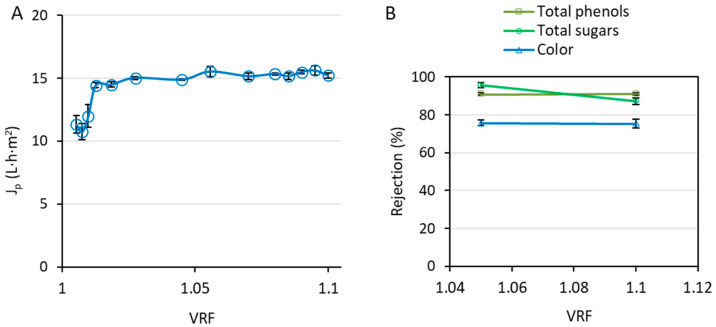
Permeate flux (**A**) and rejection of total sugar content, total solids, and color (**B**) obtained with the NF90 membrane at 1 m/s and 20 bar.

Once the phenolic compounds were purified from sugars, organic acids, free fatty acids, and triterpenes, the permeate stream (after the nanofiltration process with the NF270 membrane) was concentrated. To that end, it was submitted to a reverse osmosis process, employing the NF90 membrane. This membrane was selected because its MWCO is in the middle of a nanofiltration and a reverse osmosis process. Considering the presence of the organic solvent in the feed, the highest MWCO possible was aimed, without ignoring the high retention requirements. According to the literature, its pore size was tight enough to display high rejections of phenolic compounds [27,31]. Therefore, a high flux, as well as a high retention of solutes, was intended to be obtained with the NF90 membrane. It presented an ethanol/water 50:50 (*v*/*v*) permeability of 0.87 L·h^−1^·m^−2^·bar^−1^. The permeate flux displayed when the permeate of the NF270 membrane was treated is shown in Figure 6A.

Regarding the retention of solutes performed by the NF90 membrane (Figure 6B), satisfactory, high values were achieved.

**Figure 7:**
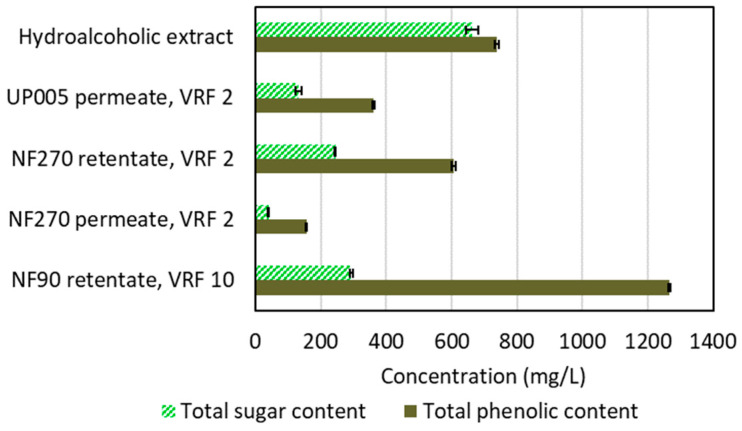
Total phenolic content and total sugar content determined in the hydroalcoholic extract of wet olive pomace, ultrafiltration permeate (UP005, 1.8 m/s, 2.5 bar), nanofiltration retentate and permeate (NF270, 1 m/s, 15 bar), and reverse osmosis retentate (NF90, 1 m/s, 20 bar). The concentration in the NF90 retentate was estimated according to Equation (3). The volume reduction factor (VRF) of each process is detailed in the figure.

Also, Figure 7 shows the total phenolic content and total sugar content in each stream implied in the integrated membrane process, revealing that, in the final stage of reverse osmosis, a high concentration of phenolic compounds, at a higher purity than in the initial hydroalcoholic extract, can be achieved.

## 3. Discussion

As can be seen in Table 1, the hydroalcoholic extract of wet olive pomace contains a remarkable concentration of valuable phenolic compounds, which were the molecules of interest in this work. Apart from those compounds, a high concentration of organic matter was present in the extract, as reflected by the high sugar and total solids content. The brownish color of the extract was reflected by a high color coefficient in comparison with other olive-derived wastewaters [32]. The slightly acidic pH was attributed to the presence of organic acids, as presented in Figure 3 and Figure 5. Regarding conductivity, the obtained values were expected, according to previous results [23], and were due to the solution of salts in the solvent.

Therefore, the application of an integrated membrane process to separate the phenolic compounds from the rest of the undesirable matter of the extract has been proposed in this work.

The first stage of the proposed treatment entailed an ultrafiltration process. As shown in Figure 1, the UF010104 membrane exhibited much higher values of permeate flux than the UP005 membrane because of a higher pore size. The high, initial permeate flux obtained with the UF010104 membrane experienced a progressive decline that continued until a volume reduction factor (VRF) of 1.7 was achieved. At the beginning of the process, flux decline was sharper, prompted by membrane fouling. After the VRF of 1.7, a steady state was reached, and the permeate flux stabilized at 20.4 ± 0.7 L·h^−1^·m^−2^. The feed solution was a real vegetable extract, with a high organic load and high content of total solids (Table 1). Furthermore, an organic solvent, whose greater viscosity (in comparison with water) reduces the flux, was present. Therefore, the performance of the UF010104 membrane was considered very productive. In the case of the UP005 membrane, the permeate flux was logically lower. An initial sharp decrease was also observed for this membrane. Due to the lower values of permeate flux displayed by this membrane, the ultrafiltration of the total volume of the hydroalcoholic extract of wet olive pomace could not be completed within the same working day. Therefore, at the end of each working day, the membrane was rinsed (as commented in Section 4.2), and the process was resumed the next day. Also, at the end of the third working day, the UP005 membrane was submitted to a cleaning process, aiming to recover the higher permeate flux that was obtained at the beginning of the ultrafiltration process. After the Ultrasil cleaning (see Section 4.2), the recovery of the solvent permeability was only 70%, in comparison with that of the new membrane, which indicates the presence of residual fouling. For that reason, the permeate flux at a VRF of 1.35 was lower than that observed at the beginning of the process (8.4 ± 0.4 L·h^−1^·m^−2^). Then, the cake layer formation started again, motivating the shape of the curve that is reflected in Figure 1. After a VRF of 1.7, the permeate flux of the UP005 membrane became more stable and, finally, it was constant at a VRF of 1.8 (4.1 ± 0.3 L·h^−1^·m^−2^) until the end of the process.

In the current literature, there are no previous studies dealing with the ultrafiltration of a solvent-based extract of wet olive pomace in cross-flow mode. To the best of our knowledge, the only related work regarding this topic is a previous study from our research group, consisting of a screening of ultrafiltration membranes in a bench-top, stirred cell to treat the hydroalcoholic extract of wet olive pomace [29]. In that study, the UF010104 and UP005 membranes were also tested, but they displayed much lower values of permeate flux than those obtained in this work, whereas the solvent permeability was similar. In fact, in this work, a permeate flux (when treating the hydroalcoholic extract of wet olive pomace) increment of 43% and 24% was obtained for UF010104 and UP005, respectively, with respect to the values obtained in the stirred cell. This was due to the effect of the tangential flow, which contributed to reduce the concentration polarization in the membrane module. As permeate flux was higher for the UF010104 membrane, the formation of the gel layer was also more relevant for this membrane, as more solutes were driven towards the membrane surface. Therefore, the effect of the tangential flow was more notable for this membrane because the gel layer formation was reduced in the cross-flow plant and, consequently, a higher permeate flux was observed.

Regarding the rejection of undesired organic matter (Figure 2), for both ultrafiltration membranes, the rejection increased slightly with VRF as a result of membrane fouling. More stable values were observed when the steady state was achieved. As commented in Section 2.2.1, the steady state was achieved around a VRF of 1.7, both for the UF010104 and the UP005 membranes. Both membranes presented high rejections of color, total solids, and total sugars. Most of the carbohydrates present in the hydroalcoholic extract of wet olive pomace are expected to be monomers and dimers, such as glucose, fructose, and sucrose [33]. Therefore, moderate rejections of total sugars were expected. Nevertheless, several aspects influenced the increment in the rejection of these compounds. First, the hydroalcoholic extract of wet olive pomace was obtained under the application of ultrasounds, and the cavitation bubbles favor the disaggregation of the cell wall and contribute to a higher recovery of the targeted phenolic compounds. In this process, some oligosaccharides can be released [34,35]. These are larger compounds (with respect to simple sugars), which can be more easily rejected by ultrafiltration membranes. Secondly, the complexity of the wet olive pomace extract is undeniable (Table 1), as a high content of organic matter is present. Among them, sugars and phenolic compounds contain functional groups (such as hydroxyl groups and acid groups) susceptible of reacting between each other [36,37]. As a consequence, the potential complexation between these different molecules would increase the size of the solutes and increase their retention. Thirdly, the presence of this organic solvent (ethanol/water 50:50 *v*/*v*) has previously been demonstrated to reduce the hydrophilicity of UF010104 and UP005 membranes [29]. In this scenario, a reduction in the affinity of sugars (which are polar molecules) and the active layer of the membranes also supports a reduction in the permeation of these compounds. Finally, in the case of the UP005 membrane, it is a tight ultrafiltration membrane, which also contributes to the increase in the rejection of sugars.

In general, the lower MWCO of the UP005 membrane favored higher rejections. At a VRF of 2, the UP005 membrane displayed a rejection of 98 ± 3%, 64.9 ± 0.4%, and 77 ± 5%, for color, total solids, and total sugars, respectively. Despite the lower values of permeate flux achieved by the UP005 membrane (in comparison with the UF010104 membrane), the selectivity was considered a priority. In that scenario, the UP005 membrane was considered to be more suitable for the purpose of purifying the biophenols. Therefore, the determination of the phenolic compounds in the streams derived from the UP005 membrane was conducted.

As shown in Figure 3, the UP005 membrane displayed a low rejection of the phenolic compounds of interest. Only some phenolic compounds were rejected above 50%, and all of them belonged to the chemical family of secoiridoids. Among them, there were glycosylated compounds and other derivatives with a higher molecular weight. This is the case of elenolic acid glucoside, acyclodihydroelenolic acid hexoside, oleuropein (which contains a residue of glucose in its structure), and the derivative of oleuropein aglycone. Due to their chemical structure, these molecules were inevitably separated in the retentate stream. However, the most valuable compounds of the extract were the simple phenols (including the appreciated hydroxytyrosol [38,39]) and the phenolic acids, which are commonly found in cosmetic preparations [40]. Both chemical classes of simple phenols and phenolic acids were recovered in the UP005 permeate, which ensured the effectiveness of the integrated process.

In a previous work by the authors, the rejection of phenolic compounds during the ultrafiltration of aqueous extracts (without ethanol) of wet olive pomace was reported, employing the same membrane (UP005) in cross-flow mode [41]. If those results are compared with this work, it is noticeable that lower rejections of biophenols are obtained when the extract is solvent-based. In a non-aqueous, organic media, as is the hydroalcoholic extract of wet olive pomace, the polymers of ultrafiltration membranes can suffer a pore enlargement as a result of the solvent contact, leading to a decrease in rejection [26,29], as was observed here.

Regarding the compounds from the olive minor fraction analyzed by LC-MS that were not polyphenols, organic acids were more efficiently rejected than simple phenols and phenolic acids, contributing to the adequacy of the purification process. On the contrary, triterpenes and free fatty acids passed through the UP005 membrane, and their retrieval had to be addressed in the subsequent nanofiltration stage. 

As illustrated, the ultrafiltration process permitted the elimination of unwanted organic matter. However, the permeate stream still contained total solids and sugars susceptible to be withdrawn. Furthermore, the fractionation of the wide range of phenolic compounds that passed to the permeate was intended.

Therefore, the following stage of the process, a nanofiltration stage, was implemented. The flux decline observed during the nanofiltration stage (Figure 4A) was much softer than that observed during the ultrafiltration process. This is because the feed solution in this case was cleaner. After the treatment of the extract of wet olive pomace by ultrafiltration, the total solids content was largely reduced (Figure 2), which diminished the concentration polarization in the nanofiltration module. Thus, permeate flux reached a stabilized performance faster. The initial flux decline took place until a VRF of 1.13 was achieved. Afterwards, only a small decrease in the permeate flux occurred, until a steady state was reached at a VRF of 1.7, with a permeate flux of 50.2 ± 0.2 L·h^−1^·m^−2^.

The unwanted organic matter present in the hydroalcoholic extract of wet olive pomace included total sugar content, total solids content, and pigments responsible for the color of the stream. According to Figure 4B, the rejection of total solids was very stable during the whole process, surpassing 50%. When it comes to the specific determination of sugars, the rejection of total sugar content increased with the VRF, reaching a value of 75 ± 1%. Considering the aim of this work, consisting of the recovery of purified phenolic compounds, this high rejection value was greatly satisfactory. One of the main challenges of isolating phenolic compounds from agri-food products (and by-products) is their separation from sugars. The molecular weight of some carbohydrates, especially monosaccharides, such as glucose and fructose, is not far from the molecular weight of some phenolic compounds [42]. Therefore, it is a challenge to obtain the operating conditions (and select the proper membrane) to achieve this separation. Regarding color rejection, it also increased during the process, until a rejection value of 87 ± 3% was achieved. As a result, a clean, transparent stream was obtained as the permeate of the nanofiltration process.

As commented before, the aim of this work was to recover the highest possible proportion of purified phenolic compounds. This implied the reduction of the organic load, as reflected in Figure 2, but also the elimination of organic acids, triterpenes, and free fatty acids. Some of these compounds, mainly triterpenes and free fatty acids, could not be eliminated by ultrafiltration. As can be seen in Figure 5, the rejection of these undesired chemical families surpassed 75% after the nanofiltration process. Furthermore, the NF270 membrane provided an efficient fractionation of phenolic compounds. The compounds of larger size, including secoiridoids, lignans (hydroxypinoresinol), and flavonoids, were rejected in higher percentages, even reaching 100% for some compounds, and surpassing 80% in many cases. Vieira et al. also reported an almost complete retention of high-molecular-weight anthocyanins during the nanofiltration (with the NF270 membrane) of a jussara extract containing 70% (*v*/*v*) of ethanol [43].

On the contrary, Figure 5 shows that the family of phenolic acids and aldehydes was rejected to a lesser extent. The low rejection of the phenolic acids constituted one of the main achievements of this work. A complete passage of these compounds could not be expected because of the adsorption on the membrane surface, as demonstrated in a previous work by the authors [30]. In any case, the results indicate an efficient recovery of phenolic acids, as the molecules of vanillic and ferulic acids were rejected at 8.8 ± 0.5% and 35.7 ± 0.5%, respectively. The impact of phenolic acids on a consumer’s health mainly relies on their antioxidant activity (by scavenging peroxide radicals, hydroxyl radicals, etc.) [44]. Also, the molecule of vanillic acid has been observed to reduce the neuronal apoptosis related to Alzheimer’s disease [45]. Furthermore, ferulic acid supplementation has been demonstrated to improve type 2 diabetes by reducing the glucose concentration in blood [46].

Regarding simple phenols, most of them were obtained in the retentate stream. However, the observed rejection of hydroxytyrosol was 38.6 ± 0.6%, being preferentially recovered in the permeate, together with phenolic acids. This was a favorable result, considering that hydroxytyrosol is one of the most valuable phenolic compounds from the olive fruit due to its high antioxidant power [47]. It activates the expression of the transcription factor Nrf2, which determines the expression of antioxidant enzymes [48]. Also, one of its many interesting properties is the capacity to surpass the blood–brain barrier and reduce the aggregation of tau protein, which results in the prevention of Alzheimer’s disease [49].

According to the results from Figure 5, the fractionation of the phenolic compounds was achieved, obtaining a permeate stream enriched in phenolic acids and hydroxytyrosol, with numerous applications of high impact on the health of potential consumers. Afterwards, this stream was concentrated by reverse osmosis.

The interest of the NF270 permeate is undeniable considering its composition. However, the importance of the retentate stream should not be underestimated. After the nanofiltration process, simple phenols, secoiridoids, lignans, and flavonoids were concentrated in the retentate. Even though the purity of this stream is not as high as the NF270 permeate, its utilization is still possible in a final application in which the presence of sugars, triterpenic acids, and fatty acids is not detrimental. This could be the case for animal feeds. The enriching of animal feeds with a vegetable concentrated stream such as the NF270 retentate could contribute to preserving the quality of the product, as the phenolic compounds reduce the oxidation of nutrients [16]. Also, some benefits in animal health, derived from the antimicrobial and antioxidant character of the phenolic compounds, could be derived. In fact, the use of olive pomace for poultry feeding has recently been proposed [50].

Since the nanofiltration permeate was a diluted stream containing valuable phenolic compounds, it was subsequently concentrated by reverse osmosis. The evolution of permeate flux with the VRF (Figure 6A) suggested that fouling was not remarkable because it did not decrease. After being treated by ultrafiltration and nanofiltration, the hydroalcoholic extract of wet olive pomace had a low total solids content. Therefore, the concentration polarization phenomenon was not relevant. Instead of decreasing with the VRF, the permeate flux increased during the first moments of the reverse osmosis process until a VRF of 1.02 was achieved, reaching 15.3 ± 0.3 L·h^−1^·m^−2^. This can be related to the swelling of the membrane as a result of solvent contact. Several authors have reported the swelling of the NF90 during the filtration of an organic solvent [51,52]. In fact, Tamires Vitor Pereira et al. employed the NF90 membrane to treat a hydroalcoholic extract of grape marc after a pre-treatment by microfiltration. They also observed an initial increment of the permeate flux and attributed it to the polymer swelling due to the contact with ethanol. As a result of the membrane swelling, the cross-linking of the polymer might be affected, resulting in a higher distance between the polymer chains and, in consequence, a higher pore size [53].

As can be seen in Figure 6B, the membrane was able to retain the phenolic compounds above 90%. Thus, the concentration of these compounds was accomplished (as presented in Figure 7), after their purification by ultrafiltration and their fractionation by nanofiltration. The hydroalcoholic permeate stream obtained with this membrane can be reused for the rinsing of the ultrafiltration membranes during the second stage of the integrated process.

## 4. Materials and Methods

### 4.1. Extraction of Phenolic Compounds from Wet Olive Pomace

The wet olive pomace employed in this study was obtained from a two-phase olive mill in Segorbe (Castellón, Spain). The phenolic compounds were extracted from wet olive pomace using a 50% (*v*/*v*) mixture of ethanol/water as solvent. The applied methodology was previously optimized [23]. It entailed an ultrasound-assisted extraction (UAE) performed at 37 kHz and 40 °C for 45 min. In short, 800 g of wet olive pomace was dissolved in a 1:10 proportion with the organic solvent and subjected to UAE. Afterwards, the sample was centrifuged at 17,200 RCF for 6 min, and the extract was vacuum filtered with a 60 μm filter (Fanola, Barcelona, Spain). Afterwards, the resulting extract was treated by an integrated membrane process.

### 4.2. Integrated Membrane Process

**Figure 8:**
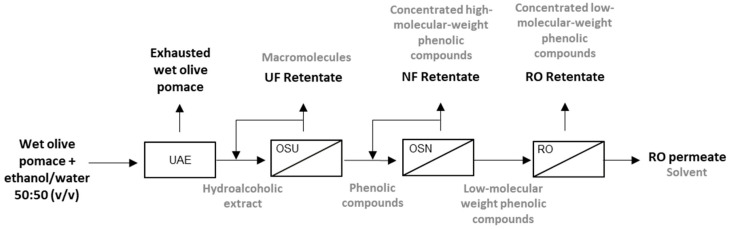
Solvent-based, integrated membrane process to purify and concentrate phenolic compounds from the hydroalcoholic extract of wet olive pomace. UAE: ultrasound-assisted solid–liquid extraction; UF: ultrafiltration; OSU: organic solvent ultrafiltration; NF: nanofiltration; OSN: organic solvent nanofiltration; RO: reverse osmosis.

The membrane process that has been proposed to purify and concentrate the phenolic compounds from the hydroalcoholic extracts of wet olive pomace is reflected in Figure 8. It entails an ultrafiltration process to withdraw a high proportion of total solids and sugars, a nanofiltration process to increment the purity of the phenolic compounds and fractionate them, and a reverse osmosis process to concentrate the permeate stream obtained in the nanofiltration step. As the organic solvent was present throughout the whole process, it entailed an additional challenge regarding the performance of the membranes.

It should be noted that the retentate obtained during the nanofiltration process is also a valuable stream. As explained, the nanofiltration retentate contains a significant concentration of phenolic compounds. It was also a concentrated stream, enriched in high added-value compounds.

### 4.3. Solvent-Resistant Ultrafiltration

The obtained hydroalcoholic extract of wet olive pomace was subjected to an ultrafiltration process, employing an ultrafiltration cross-flow plant (Orelis Environment, Salindres, France) equipped with a Rayflow membrane module (Orelis Environment, Salindres, France). The module contained two ultrafiltration membranes working in series. According to this configuration, the retentate of the first membrane corresponds to the feed of the second membrane. As the permeate volume can be despised in comparison with the retentate volume, it can be assumed that both membranes treat the same feed during the ultrafiltration process. This configuration allows the testing of two membranes at the same time in order to determine which one is more suitable for the purpose of the process. Different MWCOs and materials were considered. The area for each membrane was 129 cm^2^. The information regarding the membranes tested in this work can be found in Table 2.

Prior to the ultrafiltration of the hydroalcoholic extract, the membranes were immersed for two hours in a solution of ethanol/water 50:50 (*v*/*v*) to hydrate them and remove any conservative remnants. Afterwards, the UF010104 and the UP005 membranes were compacted (using a solution of ethanol/water 50:50 (*v*/*v*) as feed) at a transmembrane pressure (TMP) of 2.2 bar and 2.7 bar, respectively. The solvent permeability ($L_{p}$) of the membranes was investigated, in the range of 0.75–2 bar for the UF010104 membrane and 1–2.5 bar for the UP005 membrane. The solvent permeability was calculated in terms of the permeate flux ($J_{p}$) and the transmembrane pressure according to the following equation:

(1) $$L_{p}=\frac{J_{p}}{TMP}$$

Subsequently, the extract was ultrafiltered at 2 bar for the UF010104 membrane and at 2.5 bar for the UP005 membrane, with a cross-flow velocity of 1.8 m·s^−1^ and 25 °C. These operating conditions were selected according to previous studies from our research group [23,29]. The process was carried out in concentration mode until a VRF of at least 2 was achieved. Samples from retentate and permeate streams were collected at different VRFs to analyze the efficiency of the process and calculate rejection values.

If the ultrafiltration process was not completed within the same working day, the membrane was rinsed and the process was resumed the next day. This rinsing consisted of flushing the ultrafiltration plant with a solution of ethanol/water 50:50 (*v*/*v*) for 15 min. The solvent employed daily for the membrane rinsing was not directly discarded. On the contrary, the solution was recovered from the plant and reused for three days in order to contribute to the sustainability of the process and reduce the costs.

When necessary, the UP005 membrane had to be cleaned. Then, an Ultrasil 110, at 1% in water (*v*/*v*), was employed after rinsing the membrane with the solvent mixture for 15 min. The cleaning solution was recirculated through the plant for 1 h at 35 °C, working at 0.7 bar and 1.8 m·s^−1^. Later, the membrane was rinsed with tap water to remove the Ultrasil solution. Finally, a rinsing with ethanol/water 50:50 (*v*/*v*) was applied. In the case of UF010104, it was not necessary to insert a cleaning stage during the ultrafiltration process developed to treat the wet olive pomace extract.

### 4.4. Solvent-Resistant Nanofiltration

The ultrafiltration permeate was treated in a nanofiltration cross-flow plant equipped with a stainless steel module specifically designed for the plant [54]. The membrane area was 72 cm^2^. A NF270 (Microdyn Nadir, Wiesbaden, Germany) membrane was used for this process. This membrane was selected according to previous results from our research group, focused on the same application [30].

Prior to the nanofiltration stage, the membrane was immersed overnight in ethanol/water 50:50 (*v*/*v*) as a preconditioning treatment. Afterwards, a compaction stage was carried out at 22 bar and a cross-flow velocity of 1 m·s^−1^ until a stable permeate flux was observed. The solvent permeability was calculated using Equation (1) in a range of 5–20 bar at 1 m·s^−1^.

Afterwards, the ultrafiltered extract was nanofiltered at 15 bar and 1 m·s^−1^, in concentration mode as well. Samples from both retentate and permeate streams were collected at different VRF values for further characterization.

### 4.5. Solvent-Resistant Reverse Osmosis

The previously obtained nanofiltered permeate was treated as the feed in a reverse osmosis process, which served as a final stage to concentrate the phenolic compounds. The set-up implemented was similar to the nanofiltration plant (Section 2.3), but, in this case, a NF90 membrane was selected for the process. 

As in the previous stages of the integrated process, preconditioning and compaction of the membrane were necessary before the experiment. The NF90 membrane was compacted at 22 bar and 1 m·s^−1^, and the solvent permeability was studied under the same conditions as in the nanofiltration step. The reverse osmosis process was carried out with the nanofiltered permeate at 20 bar and 1 m·s^−1^.

### 4.6. Analysis of the Streams

#### 4.6.1. Analysis of Organic Matter

Samples obtained from the feed extract, retentate, global, and instantaneous permeates from all the separation process were characterized in terms of color, total solids, total sugars, and total phenolic content, as well as pH and electric conductivity. Each sample was analyzed at least in duplicate, and the instantaneous rejection of each solute (*R*) was calculated according to Equation (2):

(2) $$R=(1-\frac{C_{p}}{C_{r}})\cdot 100$$

where *C_p_* is the concentration in the instantaneous permeate collected at a given VRF, and *C_r_* represents the concentration in the retentate stream.

In the case of the reverse osmosis process, the concentration of total phenolic content and total sugars was estimated at a VRF of 10. This VRF was selected according to previous results dealing with the reverse osmosis concentration of a hydroalcoholic stream derived from the agri-food sector, employing a polyamide membrane [55]. To estimate the concentration of total phenolic content and total sugars at a VRF of 10, Equation (3) was used:

(3) $$C_{r}=C_{o}\cdot VRF^{R}$$

where $C_{r}$ represents the concentration in the retentate stream, $C_{o}$ represents the concentration in the feed solution (which corresponds to the nanofiltration permeate in this case), VRF is the volume reduction factor, and R is the rejection coefficient, which was considered to be constant during the reverse osmosis process. This assumption was supported by the results presented in Figure 6B. The equation is derived from a mass balance under non-stationary conditions [56].

Color was assessed according to UNE-EN ISO 7887:2012 [57], measuring the absorbance at 436 nm, 525 nm, and 620 nm with a UV-VIS DR 6000 spectrophotometer (Hach Lange, Ames, IA, USA. The color coefficient was calculated with the following formula:

(4) $$Colour=\frac{A_{436}^{2}+A_{525}^{2}+A_{620}^{2}}{A_{436}+A_{525}+A_{620}}$$

The Folin–Ciocalteu methodology was applied to determine the concentration of total phenolic compounds [58]. To quantify the analytes, tyrosol (VWR International, Radnor, PA, USA) was employed to prepare the calibration curve, diluted in ethanol/water 50:50 (*v*/*v*) in the range of 1–500 mg·L^−1^. Total sugar content was determined via the anthrone method [59], employing a glucose (Roche, Basel, Switzerland) calibration curve, prepared in ethanol/water 50:50 (*v*/*v*) in the range of 1–125 mg·L^−1^. Total solids were obtained by evaporation of a known aliquot of the sample. A pHmeter (GLP21+, Crison, Barcelona, Spain) and a conductimeter (GLP31+, Crison, Spain) were used to measure the pH and conductivity of the samples.

#### 4.6.2. Characterization of the Olive Minor Fraction

To determine the analytes belonging to the olive minor fraction, an analytical methodology based on liquid chromatography (LC) coupled to mass spectrometry (MS) was applied. The employed instrument was an Agilent 1260 Infinity II liquid chromatograph coupled to a 6546 quadrupole-time-of-flight (QToF) mass analyzer equipped with electrospray ionization (ESI) (Agilent Technologies, Santa Clara, CA, USA). A Zorbax Extend C18 column (4.6 × 100 mm, 1.8 µm) (Agilent Technologies, USA) was used to separate the compounds, operating at 40 °C and a flow rate of 0.9 mL/min after a sample injection of 4 µL. Ultrapure water (Direct-Q^®^ 3UV system, Merck Millipore, Darmstadt, Germany) and LC-MS grade acetonitrile (Honeywell, Wabash, IA, USA) were employed as phase A and phase B, respectively. Both phases were acidified with 0.5% pure acetic acid (VWR International, Radnor, PA USA). The separative conditions and the specific parameters for the mass spectrometry were previously developed [23]. A semi-quantitative approach was performed, conducting an external calibration with pure standards of citric acid (VWR International, Radnor, PA, USA), tyrosol (VWR International, Radnor, PA, USA), hydroxytyrosol (Sigma Aldrich, Darmstadt, Germany), caffeic acid (VWR International, Radnor, PA, USA), *p*-coumaric acid (Sigma Aldrich, Darmstadt, Germany), oleuropein (Sigma Aldrich, Darmstadt, Germany), luteolin (VWR International, Radnor, PA, USA), decarboxymethyl oleuropein aglycone (Sigma Aldrich, Darmstadt, Germany), and hydroxy-octadecanoic acid (Sigma Aldrich, Darmstadt, Germany). Standard solutions were prepared in the range of 0.1–100 mg·L^−1^.

## 5. Conclusions

An integrated membrane process to purify and concentrate the phenolic compounds present in hydroalcoholic extracts of wet olive pomace was developed, aiming to obtain natural phenolic compounds with interesting health benefits, such as antioxidant, antidiabetic, and neuroprotective capacities. First, the phenolic compounds were extracted by an ultrasound-assisted solid–liquid extraction, which was performed with ethanol/water 50:50 (*v*/*v*). Therefore, all the subsequent stages occurred in organic media. The extract was treated by ultrafiltration in order to reduce the total solids content and separate the phenolic compounds from sugars. For this step, the UF010104 and the UP005 membranes were tested. The UP005 membrane displayed a higher rejection of the unwanted compounds, whereas the passage of phenolic compounds was high. Therefore, this membrane was preferred. Once the phenolic compounds were purified during the ultrafiltration stage, they were fractionated by means of a nanofiltration process (employing the NF270 membrane), which permitted the recovery of hydroxytyrosol and phenolic acids (such as vanillic and ferulic acids) in the nanofiltration permeate. Furthermore, additional sugars and total solids were largely retained, enlarging the purity of the permeate stream. Selecting the stream of interest during the nanofiltration process depends on the final application of the compounds. The permeate stream contained high-purity low molecular weight phenolic compounds (phenolic acids and hydroxytyrosol), which should be later concentrated, but the retentate also contained a wide range of already concentrated compounds (simple phenols, secoiridoids, lignans, and flavonoids) whose antioxidant properties are not minor.

Finally, to concentrate the permeate of the nanofiltration stage, reverse osmosis was proposed, employing an NF90 membrane. This process allowed us to recover the purified phenolic compounds at a higher concentration in the retentate, which constituted a valuable stream, enriched in high added-value compounds.

## Figures and Tables

**Table 1:** Characterization of the extract of wet olive pomace obtained with ethanol/water 50:50.

Parameter	Determined Concentration
Total phenolic content (mg/L)	737 ± 6
Total sugar content (mg/L)	663 ± 18
Total solids (g/L)	7.78 ± 0.02
Color coefficient	2.3 ± 0.1
pH	5.9 ± 0.2
Conductivity (µS/cm)	679 ± 30

**Table 2:** Characteristics of the membranes employed in this study.

Membrane	MWCO (kDa) ^1^	Material	Manufacturer	Process
UF010104	20 ^2^	Proprietary	SolSep BV	UF
UP005	5	PES ^3^	Microdyn Nadir	UF
NF270	0.3–0.4	Polyamide	DuPont	NF
NF90	0.2	Polyamide	DuPont	RO

^1^ Molecular weight cut-off. ^2^ Determined in hexane. ^3^ Polyethersulfone.

## Data Availability

The data presented in this study are available on request from the corresponding author.
